# Improved Oxygen Supply to Multicellular Spheroids Using A Gas-permeable Plate and Embedded Hydrogel Beads

**DOI:** 10.3390/cells8060525

**Published:** 2019-05-31

**Authors:** Hirotaka Mihara, Mai Kugawa, Kanae Sayo, Fumiya Tao, Marie Shinohara, Masaki Nishikawa, Yasuyuki Sakai, Takeshi Akama, Nobuhiko Kojima

**Affiliations:** 1 Department of Life and Environmental System Science, Graduate School of Nanobioscience, Yokohama City University, 22-2 Seto, Kanazawa-ku, Yokohama 236-0027, Japan; n175275c@yokohama-cu.ac.jp (H.M.); n175352c@yokohama-cu.ac.jp (K.S.); n165351e@yokohama-cu.ac.jp (F.T.); akama@yokohama-cu.ac.jp (T.A.); 2 Faculty of Science, International College of Arts and Science, Yokohama City University, 22-2 Seto, Kanazawa-ku, Yokohama 236-0027, Japan; i170239g@yokohama-cu.ac.jp; 3 Department of Chemical Systems Engineering, Graduate School of Engineering, The University of Tokyo, 7-3-1 Hongo, Bunkyo-ku, Tokyo 113-8656, Japan; marie-s@chemsys.t.u-tokyo.ac.jp (M.S.); masaki@chemsys.t.u-tokyo.ac.jp (M.N.); sakaiyasu@chemsys.t.u-tokyo.ac.jp (Y.S.)

**Keywords:** multicellular spheroids, 3D culture, gas-permeable plate, hydrogel beads, methylcellulose

## Abstract

Culture systems for three-dimensional tissues, such as multicellular spheroids, are indispensable for high-throughput screening of primary or patient-derived xenograft (PDX)-expanded cancer tissues. Oxygen supply to the center of such spheroids is particularly critical for maintaining cellular functions as well as avoiding the development of a necrotic core. In this study, we evaluated two methods to enhance oxygen supply: (1) using a culture plate with a gas-permeable polydimethylsiloxane (PDMS) membrane on the bottom, and; (2) embedding hydrogel beads in the spheroids. Culturing spheroids on PDMS increased cell growth and affected glucose/lactate metabolism and CYP3A4 mRNA expression and subsequent enzyme activity. The spheroids, comprised of 5000 Hep G2 cells and 5000 20 µm-diameter hydrogel beads, did not develop a necrotic core for nine days when cultured on a gas-permeable sheet. In contrast, central necrosis in spheroids lacking hydrogel beads was observed after day 3 of culture, even when using PDMS. These results indicate that the combination of gas-permeable culture equipment and embedded hydrogel beads improves culture 3D spheroids produced from primary or PDX-expanded tumor cells.

## 1. Introduction

Patient-derived xenograft (PDX) models are important because they can maintain tumors isolated from patients with their complex heterogeneity and molecular diversity retained [1,2,3,4]. PDXs are expected to be highly predictive in preclinical testing when compared with conventional two-dimensional culture, in which even established cell lines rapidly lose their original properties [5,6,7]. As well as PDX models, in vitro methods are becoming popular for reproducing cancer tissues in 3D microenvironments. Tumor-organoid cultures (grown from a single stem cell) are now popular for recapitulating the hierarchical structure of tumor tissues in vitro [8]. Formation of tumor spheroids (from minced tumor tissues or multiple-cell suspensions) is also an attractive option for stable cultivation of tumor tissues in vitro. Inoue and colleagues developed CTOS, which enables the maintenance of patient-derived cancer cells in a 3D culture system [9]. Organoid culture using patient-derived tumors in combination with immune cells at an air–liquid interface have also been developed [10]. Another group was utilizing perfusion-based bioreactor to maintain the tumor environment of cancer tissue [11]. These in vitro systems are indispensable for high-throughput drug screening using tumors directly isolated from patients, as well as those expanded by PDX.

The major difference between PDX and other in vitro 3D culture methods is the distribution of oxygen and nutrients in the cancer tissues. In the PDX method, xenografts contain an integrated vascular system from the recipient animal, facilitating an adequate supply of oxygen and nutrients. However, these factors are difficult to control in in vitro 3D culture methods because of the lack of blood vessels, leading to necrotic cell death in the central regions of the 3D culture. Such undesired cell death makes it difficult to grow cancer tissues/cells and observe the effects of anti-cancer drugs, especially at low doses during long-term culture. It is also problem that anti-cancer drug cannot effectively access internal cells in the conventional 3D culture methods as well as biopsy studies.

We previously developed a gas-permeable bottom plate using a polydimethylsiloxane (PDMS) sheet to improve gas exchange in 3D cultured tissue [12,13]. We also developed a method to fabricate 3D hybrid spheroids comprised of cells and with 20 µm-diameter hydrogel beads. When we mixed an equal number of cells and beads, the beads formed microchannel-like structures in the spheroids [14], which enhanced protein secretion by hepatocytes and pancreatic beta cells [14,15,16]. These effects were thought to be derived from improved oxygen and nutrient supply or waste product removal. The combination of the gas-permeable plate with the hydrogel beads method might be effective to regulate oxygen concentration inside of spheroids under the static culture condition, which allows the use of high content analyzers.

In this report, we investigated the effect of the combined use of gas-permeable plate and embedded hydrogel beads on the oxygen supply to 3D spheroids by looking at cell growth, glucose/lactate consumption/production and CYP3A4 expression/activity. The distribution of oxygen was estimated by cellular function, and we defined four zones of high-, mid-, and low oxygen pressure as well as necrotic regions. Based on our results, we discuss the differential effects of oxygen supply between gas-permeable plates and hydrogel beads. These efforts offer new approaches to modifying PDX in vitro, as well as the utilization of expanded tumor cells in PDX models for high-throughput screening.

## 2. Materials and Methods

### 2.1. Cell Culture

The human hepatoma cell line Hep G2 (Japanese Collection of Research Bioresources Cell Bank, Osaka, Japan) was obtained from the Japanese Center Research Bank and grown in Dulbecco’s Modified Eagle’s Medium (DMEM; 041-2977, FUJIFILM Wako, Osaka Japan) supplemented with 10% fetal bovine serum (s-1780-500, Biowest, Nuaillé, France) and 1% penicillin-streptomycin (168-23191, FUJIFILM Wako). Cells were incubated at 37 °C, 5% CO_2_, and 100% humidity using a CO_2_ incubator, and maintained at sub-confluency by allowing passaging every two or three days.

### 2.2. Gas-Permeable Plate

A gas-permeable PDMS sheet was attached under a bottomless 24-well plate made of acrylic resin, as reported previously [12,13]. The sheet was produced by mixing 13 g of Silpot 184 and 1.3 g of crosslinking reagent (Dow Toray, Co., Ltd., Tokyo, Japan), pouring the mixture into a square dish (36-3458, Eiken Chemical, Tokyo, Japan) to a thickness of approximately 3 mm, baking at 60 °C overnight, cutting it to the size of the bottom of the plate, and sterilizing it by ultraviolet irradiation for 15 min. After attaching the PDMS sheet to the bottom of plate with a metal plate and screws, the absence of leaks was confirmed using 70% ethanol.

### 2.3. Hydrogel Beads

An inkjet PulseInjector and WaveBuilder system (Cluster Technology, Osaka, Japan) were used to form hydrogel beads with a diameter of approximately 20 µm, as described previously [14,16]. Solutions of 1.5% sodium alginate and 5% calcium chloride were sterilized and the sodium alginate solution was loaded into the nozzle cassette while the calcium chloride solution was poured into a Petri dish and used to collect and gel the alginate droplets as they were discharged from the nozzle. The calcium chloride solution was agitated using a magnetic stirrer during this process, which used a 25 µm of nozzle pore size and a voltage of 15 V and frequency of 1000 Hz.

### 2.4. Methylcellulose (MC) Medium

To prepare 3% MC medium, 3 g of methylcellulose (M0512, Sigma-Aldrich, St. Louis, MO, USA) and a magnetic stirrer bar were placed into a glass bottle and autoclaved. One hundred ml of growth medium was then added to the bottle and stirred overnight in a cold room. Any remaining undissolved MC was broken up using a pipette and stirred until dissolved.

### 2.5. Spheroid Production

Spheroids were produced using a method described in a previous report [17] and protocol [18]. Two ml of the MC medium was poured into a 35 mm Petri dish using Microman (Gilson, Middleton, WI, USA), because of the high viscosity of the MC medium. Conventional spheroids were formed by suspending Hep G2 cells in growth medium at 5 × 10^6^ cells/mL and then injecting the suspension into the MC medium in 1 µL aliquots. This means that the number of cells per spheroid was 5000 cells. About 100 spheroids could be formed in a 35 mm dish. After 24 h, 1 mL of a 5 U/mL cellulase reagent (Onozuka RS; Yakult Pharmaceutical Industry, Tokyo, Japan) prepared in normal culture medium was added to the MC medium in the 35 mm Petri dish. The mixture was incubated for 30–60 min at 37 °C to reduce the viscosity by digesting the cellulose backbone. MC medium containing the spheroids was then transferred to a centrifuge tube and washed twice with phosphate-buffered serine (PBS) without centrifugation. The spheroids were then cultured in a 24-well plate with ultra-low attachment surface (3473, Corning, One Riverfront Plaza park, NY, USA) or gas-permeable plates for nine days in normal culture medium. The number of spheroids were adjusted five or 10 per well, and the spheroids were cultured statically. The normal culture medium was exchanged every two days. Hybrid spheroids comprising of cells and alginate hydrogel beads were produced by mixing equal volumes of a Hep G2 cell suspension (1 × 10^7^ cells/mL) and hydrogel beads (1 × 10^7^ beads/mL) in growth medium to produce a suspension of 5 × 10^6^ cells/mL and 5 × 10^6^ beads/mL. Single hybrid spheroid contains 5000 cells and 5000 beads. Subsequent processing was the same as for conventional spheroids.

### 2.6. Paraffin Sectioning and Hematoxylin-Eosin Staining

Spheroids were fixed with 4% paraformaldehyde (PFA) and 10 spheroids were suspended in 30 µL of 1.5% sodium alginate solution, which was then gelled by the addition of 30 µL of 10% calcium chloride. The gel capsules were embedded in a paraffin block and sectioned at a thickness of 6 µm. The sections were placed on a slide glass and stained using a conventional hematoxylin-eosin (HE) methodology.

### 2.7. Pimonidazole Labeling

Hypoxyprobe (HP1, Hypoxyprobe Inc., Burlington, MS, USA) was used according to the manufacturer’s instructions. Briefly, 0.58 mg of pimonidazole hydrochloride was dissolved in 10 mL of fresh culture medium and spheroids were cultured in this medium for 2 h and then immediately processed into frozen sections.

### 2.8. Frozen Sectioning and Immunostaining

Ten cultured spheroids were suspended in 30 µL of 1.5% sodium alginate solution, which was then gelled by the addition of 30 µL of 10% calcium chloride. The gels were placed into a 15% sucrose solution at 4 °C and then embedded in Tissue-Tek O.C.T. compound (Sakura Finetek Japan Co., Ltd., Tokyo, Japan) at 4 °C for 1 h before soaking with liquid nitrogen for 3 min to produce blocks for sectioning. The blocks were sectioned at a thickness of 8 µm using a cryostat (CM1950, Leica Biosystems, Wetzlar, Germany) and the sections were dried using an electric fan and then circled with PAP Pen Super-Liquid Blocker (DAIDO SANGYO, Saitama, Japan). Samples were fixed with 4% PFA, blocked with 5% skim milk reagent, labeled with 200-fold diluted anti-Ki-67 antibody (MA5-14520, Thermo Fisher Scientific, Waltham, MA) and 200-fold diluted anti-pimonidazole antibody (HP1, Hypoxyprobe) at 4 °C for overnight.

The slides were treated with 400-fold diluted Alexa Fluor 488-conjugated anti-rabbit secondary antibody to detect anti-Ki-67 antibody (Thermo Fisher Scientific), 400-fold diluted Alexa Fluor 546-conjugated anti-mouse IgG1 secondary antibody to detect anti-pimonidazole antibody (HP1, Hypoxyprobe) and 1 µg/mL of Hoechst 33,342 (346-07951, Dojindo Laboratories, Kumamoto, Japan) for 2 h. After washing, the sections were sealed with glycerol and cover slips and examined by fluorescence microscopy (BZ-X-700, Keyence Corporation, Osaka, Japan).

### 2.9. DNA Quantification

Ten spheroids from each group were collected and centrifuged at 1000× *g* at room temperature for 3 min to remove supernatants. Two hundred µL of lysis buffer (1 mM Tris, 5 mM NaCl, and 0.5 mM EDTA) was added to the spheroids and sonicated with an ultrasonic homogenizer (THU-80, AZ ONE Corporation, Osaka, Japan) to isolate genomic DNA. The treatment was performed five times for 1 sec each with minimum power. DNA concentration was measured using a QuantiFluor dsDNA System (Promega, Madison, WI, USA).

### 2.10. Specific Rates of Glucose Consumption and Lactate Production

Concentrations of glucose and lactate were analyzed using an auto analyzer (B15011-6, Xylem Inc, Rye Brook, NY, USA). Culture medium was changed at day 2 and spheroids were incubated for 24 h under the four conditions. After we collected the culture medium, spheroids were lysed, and the DNA was extracted as mentioned above. Specific rates were calculated as change of glucose or lactate concentration per DNA amount per 24 h. The specific rates were normalized with the rate of control condition.

### 2.11. RNA Extraction and cDNA Synthesis

Twenty spheroids from each condition were lysed using 250 µL of Trizol reagent (T9424, Sigma-Aldrich) in a microfuge tube and incubated for 5 min at room temperature. Fifty µL of chloroform (033-15721, FUJIFILM Wako) was then added to the sample, vortexed, and incubated for 10 min at room temperature. The microfuge tubes were centrifuged at 4 °C for 15 min at 12,000×g and 100 µL of each aqueous phase was transferred to a new microfuge tube followed by the addition of 125 µL of 2-propanol (162-17001, FUJIFILM Wako), vortexing, and incubation for 5 min at room temperature. The tubes were centrifuged at 12,000× *g* at 4 °C for 10 min, the supernatant removed, and the RNA pellet at the bottom of each tube washed with 75% ethanol (052-07221, FUJIFILM Wako). After the removal of the ethanol following centrifugation at 7500× *g* at 4 °C for 5 min, the pellet was dried and then dissolved in 30 µL of RNase-free water. RNA was quantified using a Nano Drop (ND-1000, Thermo Fisher Scientific) and then denatured by incubation at 65 °C for 5 min before being placed on ice. Reverse-transcription was performed using 8 µL RNA solution containing 500 ng of RNA and 2 µL of reagents and primers (FSQ-201, TOYOBO CO., LTD., Osaka, Japan) following the manufacturer’s instructions.

### 2.12. qPCR

Forward/reverse primers, SYBR Green I (172-5270, BIO-RAD, Bio-Rad Laboratories, Inc., Hercules, CA, USA) and cDNA were mixed in a PCR tube, and qPCR was performed using a StepOnePlus instrument (Thermo Fisher Scientific). The primers were: hCYP3A4-F (aag tcg cct cga aga tac aca), hCYP3A4-R (aag gaa gag aac act gct cgt g), hGAPDH-F (gag tca acg gat ttg gtc) and hGAPDH-R (ggc aac aat atc cac ttt ac). mRNA expression of CYP3A4 was normalized by that of GAPDH, and values were expressed as fold-change compared to the Control condition.

### 2.13. CYP3A4 Activity Assay

Ten spheroids for each condition were cultured in conventional and gas-permeable 24-well plates then washed with PBS. The spheroids were then incubated with 300 µL of growth medium containing 2500-fold diluted Luciferin-IPA (V9001, Promega) in a CO_2_ incubator (37 °C, 5%) for 6 h. Fifty µL of the incubated medium was then transferred to a 96 well plate (236107, Thermo Fisher Scientific) for luminescence detection, and 50 µL of detection reagent was added to each well followed by incubation in the CO_2_ incubator for 10 min. Luminescence was detected using a SPARK plate reader (TECAN, Männedorf, Switzerland) and CYP3A4 activity plotted as fold-change compared with the Control condition.

### 2.14. Statistical Analysis

Data were statistically analyzed by one-way ANOVA followed by Dunnett’s test, and *p* < 0.05 was considered to be statistically significant (Prism 8, GraphPad Software, San Diego, CA, USA).

## 3. Results

### 3.1. Spheroid Formation and Culture Conditions

**Figure 1:**
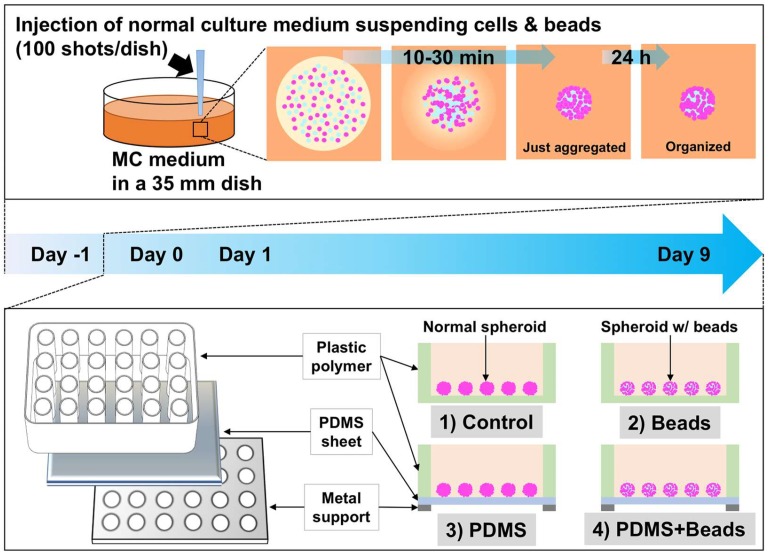
Schematic illustration of hydrogel bead-embedded spheroids and gas-permeable plate. Cells/beads suspended in normal culture medium were injected into 3% MC medium. The injected normal culture medium was adsorbed by the surrounding MC medium and the cells/beads aggregated between 10 and 30 min after injection. The aggregated cells/beads were cultured for 24 h without settling because of the high viscosity of the 3% MC medium, and 24 h was sufficient to organize the spheroids containing hydrogel beads. After 24 h, the spheroids were isolated from the MC medium using cellulase to reduce its viscosity. The conventional and bead-containing spheroids were then cultured on either normal polystyrene plates or ones with gas-permeable PDMS bottoms. Hydrogel beads and PDMS were expected to enhance oxygen supply to the spheroids.

Our approach to improving oxygen supply to spheroids was based on two factors, namely, modifying the materials of the culture plate and improving the architecture of multicellular spheroids. A gas-permeable plate with a basal PDMS sheet at the bottom of the plate was used to accomplish the former (Figure 1, lower panel). Because the solubility of oxygen in PDMS (10.6 nmol/mL/mmHg; [19]) is 8.9 times higher than in medium (1.19 nmol/mL/mmHg; [20]), cells can obtain oxygen more effectively after attaching to a PDMS sheet than they can simply receive from a culture medium [13]. The latter approach was accomplished by producing “hybrid spheroids” comprising of cells and an equal number of 20 µm-diameter hydrogel beads that had been cultured together in MC medium for 24 h (Figure 1, upper panel). Control spheroids were produced in the same way, but lacked the hydrogel beads and were used to compare with the hybrid spheroids. Both types of spheroids were isolated from the MC medium and then incubated on either ultra-low-attachment control or gas-permeable PDMS plates.

In order to investigate whether the combination of the gas-permeable plate and hydrogel beads improve oxygen supply to the centers of spheroids, we compared the four conditions (Figure 1): conventional spheroids cultured on polystyrene plates (Control), spheroids with hydrogel beads cultured on control plates (Beads), conventional spheroids cultured on gas-permeable plates (PDMS), and spheroids with hydrogel beads cultured on gas-permeable plates (PDMS + Beads). The experimental design of this study is depicted in Figure 1. Production of hybrid spheroids by the injection of a cell/bead suspension into MC medium was performed on day -1, and isolation of the spheroids from the MC medium was performed on day 0. All groups were cultured for nine days in normal culture medium, which was changed every two days.

### 3.2. Differences in Spheroid Cell Growth and Energy Metabolism

**Figure 2:**
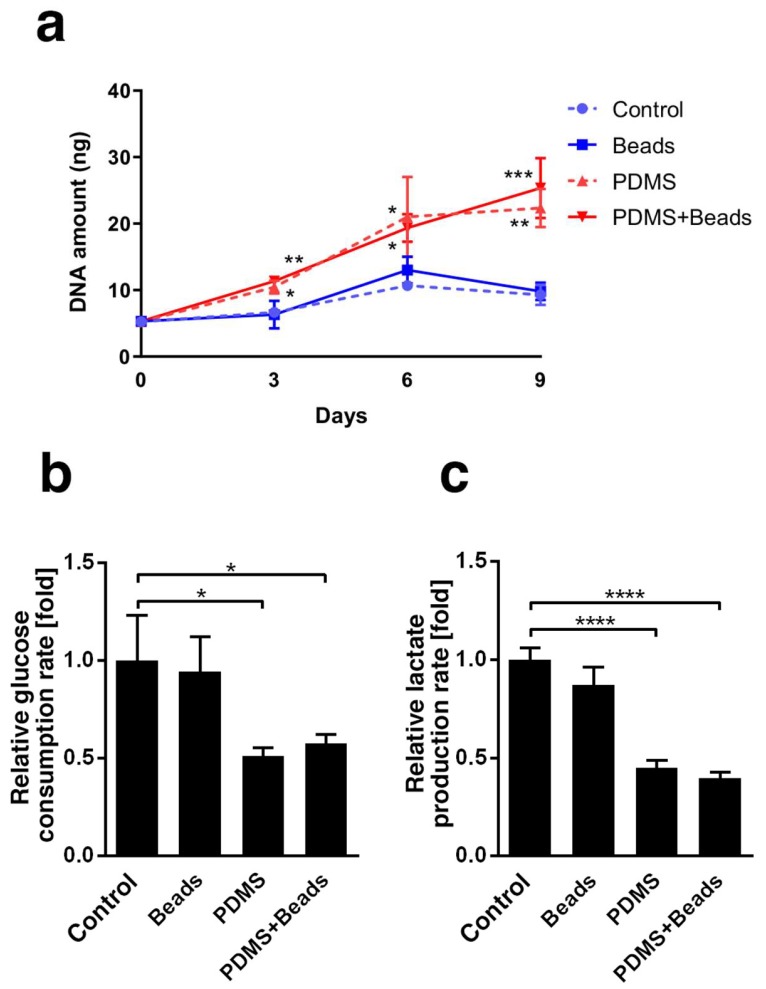
DNA content, glucose consumption and lactate production of spheroids. (**a**) DNA was extracted from 10 spheroids and measured at days 0, 3, 6 and 9. Blue and red represent polystyrene or gas-permeable conditions, respectively. Broken and solid lines represent normal or hydrogel bead-embedded spheroids. (**b**) Glucose consumption rate was calculated at day 3 and expressed as fold-change relative to Control condition. (**c**) Lactate production rate was calculated at day 3 using the same samples as for glucose consumption. * *p* < 0.05, ** *p* < 0.01, *** *p* < 0.001, **** *p* < 0.0001.

Genomic DNA was extracted from the spheroids and analyzed to determine the effect of culture conditions on cell growth. The DNA content under each condition were almost the same at day 0 and increased over time (Figure 2a). DNA content under the PDMS and the PDMS + Beads conditions was clearly higher than under the Control and the Beads conditions, indicating that oxygen supply from the gas-permeable plate bottom improved cell proliferation. In contrast, the presence of beads did not affect DNA content in either polystyrene or gas-permeable plate. Oxygen supply rates could change cellular metabolism, so we also assessed whether culture conditions affected glucose consumption and lactate production. Glucose consumption and lactate production almost halved under the PDMS and PDMS + Beads conditions compared with the Control and Beads conditions at day 3 (Figure 2b,c); however, there was no significant difference between the Control and Beads conditions or between the PDMS and PDMS + Beads conditions. These data suggest that the dominant factor affecting glucose consumption and lactate production was the presence or absence of a gas-permeable plate, since efficient ATP production by oxidative phosphorylation might reduce both glucose consumption and lactate production.

### 3.3. Enhancement of CYP3A4 Gene Expression and Enzyme Activity

**Figure 3:**
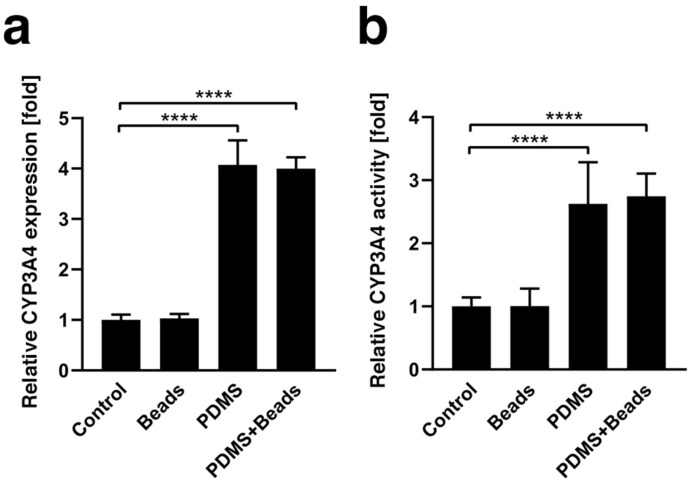
Gene expression and activity of CYP3A4. (**a**) CYP3A4 mRNA expression under Control, Beads, PDMS and PDMS + Beads conditions was measured by qPCR using spheroids at culture day 1. The results were normalized by the expression of GAPDH mRNA and are presented as the fold-difference compared with that of the Control condition. (**b**) CYP3A4 enzyme activity under each condition was measured at day 1 and is presented as the fold-change compared with the Control condition. **** *p* < 0.0001.

CYP3A4 gene expression was measured at day 1 (Figure 3a) and found to be approximately four times higher under the PDMS and PDMS-Beads conditions than the Control and Beads conditions, and there was no significant difference between hybrid and conventional spheroids regardless of the use of polystyrene or gas-permeable plates. CYP3A4 enzyme activity was also evaluated at day 1 and showed trends similar to those of gene expression (Figure 3b). These data suggest that the gas-permeable membrane plate enhances CYP3A4 expression and activity.

### 3.4. Prevention of Spheroid Core Necrosis

**Figure 4:**
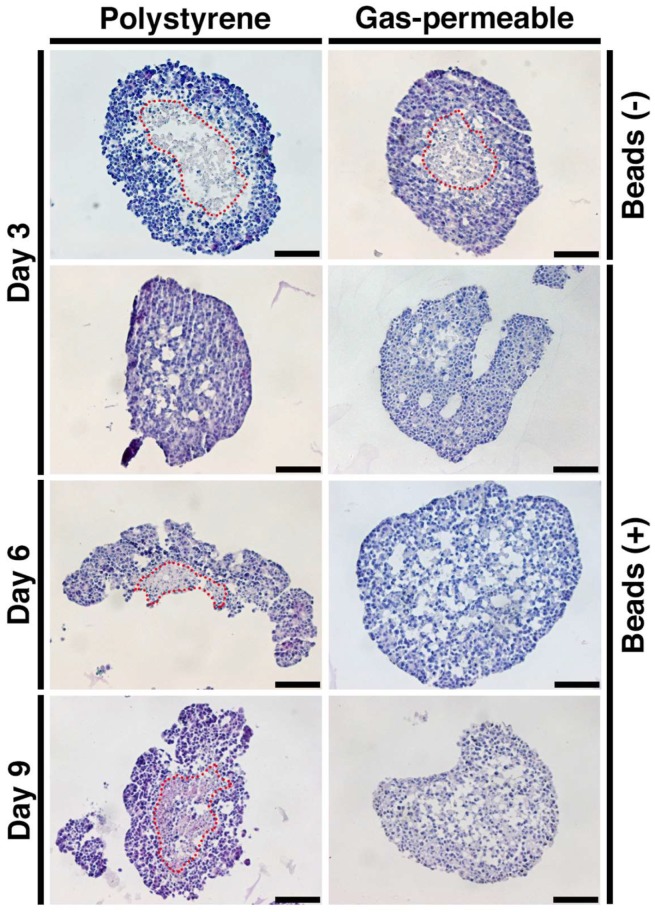
HE staining of spheroids. Spheroids at day 3, 6, and 9 and under each condition were fixed, sectioned and stained with HE. Red broken lines delineate borders between healthy and necrotic areas. Scale bars: 100 µm.

The morphology of spheroids was examined using HE staining after three, six or nine days of culture under the four conditions, and typical necrotic cores were detected at day 3 under the Control and PDMS conditions (Figure 4). In contrast, cores remained healthy under the Beads and PDMS + Beads conditions, although cores under the Beads condition began exhibiting necrosis at day 6 with the necrotic spheroids breaking into several pieces. Only the PDMS + Beads condition prevented cell death of the center of the spheroids over the entire nine days, suggesting that the effects of the combination of gas-permeable plate and the hydrogel beads on oxygen supply was effective and additive with respect to the prevention of core necrosis.

### 3.5. Spheroid Oxygen Distribution

Oxygen distribution within the spheroids was visualized by treating conventional and hybrid spheroids at day 1 with a culture media containing pimonidazole hydrochloride for 2 h to label hypoxic areas. Frozen sections of the spheroids were used to detect nuclei, pimonidazole accumulation and Ki-67 protein. Hoechst 33342 (Hoechst) stains the nuclei of living, dying or recently dead cells, but not those that have been dead for a substantial time. Therefore, Hoechst-signal-negative areas in spheroids represent necrotic cores that were deprived of oxygen. In contrast, pimonidazole forms at an oxygen pressure of 10 mm Hg [21,22], which is lower than that needed for HIF-1 expression [23], while Ki-67 protein is expressed by growing cells [24], and so serves as a marker of cells under relatively higher oxygen tension. Spheroid regions were divided into four possible areas based on these signals: necrotic (Hoechst-/Pimonidazole-/Ki-67-) and low- (Hoechst+/Pimonidazole+/Ki-67-), mid-(Hoechst+/Pimonidazole+/Ki-67+), and high-oxygen (Hoechst+/Pimonidazole-/Ki-67+).

**Figure 5:**
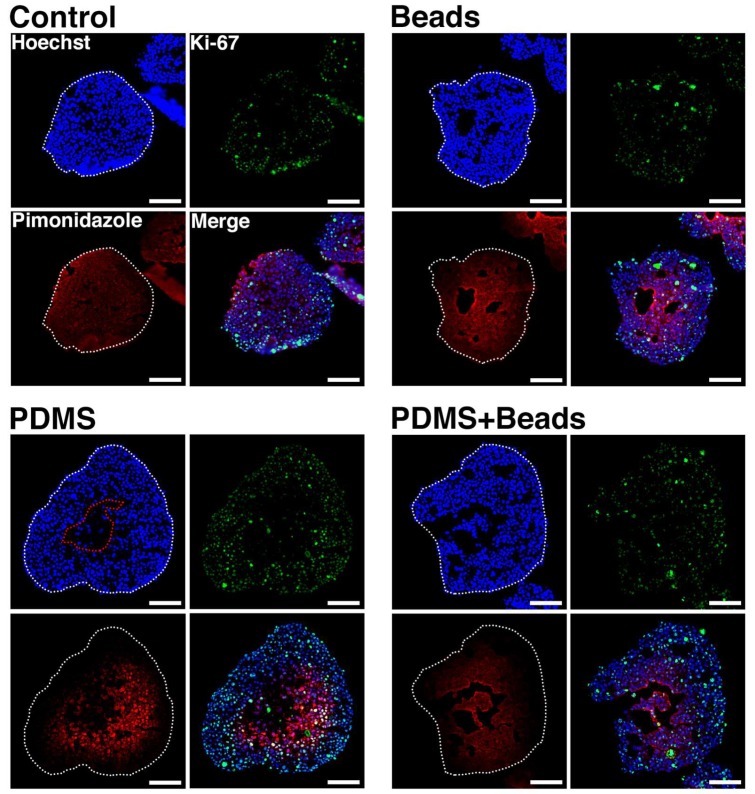
Nuclear, Ki-67 and pimonidazole visualization within spheroids. Frozen sections from spheroids at day 1 were stained with Hoechst (blue), anti-Ki-67 antibody (green), and anti-pimonidazole antibody (red). White broken lines indicate the outlines of the spheroids. Scale bars: 100 µm.

Figure 5 depicts spheroid sections at day 1 under each condition. Necrotic areas were observed under the PDMS condition, but not the others, at this time point. Low-oxygen areas were indistinct under all conditions because Ki-67-positive cells were widely distributed, indicating that there were thick regions of mid-oxygen areas under all conditions. High-oxygen areas were clearly detected under both PDMS and PDMS + Beads conditions, but they were rare under the Control and Beads conditions. Peripheral areas were pimonidazole negative as well as highly Ki-67-positive under the PDMS and PDMS + Beads conditions, indicating a clear effect of the gas-permeable membrane. The PDMS condition exhibited a necrotic core compared with the Control condition, which may have been due to elevated peripheral cell growth. Embedding of hydrogel beads was also effective in preventing necrotic core formation when combined with the use of the gas-permeable membrane.

## 4. Discussion

In this report, we attempted to improve oxygen supply to multicellular spheroids, which have the potential to be used for drug high-throughput screening using tumor cells from patients and PDX systems. Our strategy was to combine both gas-permeable culture plates and hydrogel bead-embedding into the spheroids. To judge the effect of oxygen supply, we opted to use spheroids containing 5000 cells, because such spheroids having a diameter of over 300 µm produce central necrotic cores after three days of culture. The data presented in this report support the validity of our concept for improving the culture of spheroids. It is also possible to form spheroids with diameters smaller than 100 µm, and such smaller spheroids could further extend necrosis-free culture periods, as well as enhance cellular metabolic functions.

Since many cancers progress via the infiltration of blood vessels, oxygen supply is a critical factor in their pathology [25,26]. A two-dimensional plate culture is the easiest way to maximize gas exchange in vitro, however it does not recapitulate other microenvironments for tumor maintenance [27]. Spheroid culture, which has the potential to form microenvironments, results in a reduced oxygen supply to the central part of the cell mass [28], leading to a need to improve the oxygen supply without losing features of the microenvironment. PDMS has excellent gas-absorbing properties and can dissolve 8.9 times more oxygen than culture medium. Therefore, a culture dish with a PDMS bottom could potentially supply a higher oxygen partial pressure to cells in contact with the PDMS than a conventional dish could [13]. In addition, Hamon et al. demonstrated that the oxygen supply from a PDMS sheet resulted in the formation of 130 µm-thick multilayer cell structures [29].

The effect of the gas-permeable bottom was clear in this study. In addition to our observation on the growth of Hep G2 spheroids (Figure 2a), it has been reported that rat primary hepatocytes and mouse fibroblast cell lines cultured on PDMS sheets grow faster than on control polystyrene plates [12]. The decrease in glucose consumption (Figure 2b) and lactate production (Figure 2c) observed in this study may have been due to the efficient production of ATP by aerobic culture rather than through anaerobic pathways, such as glycolysis. Enhancement of CYP3A4 mRNA expression observed here (Figure 3) was comparable to that in previous reports, for example, where a dynamic perfusion culture that could supply oxygen at higher level also enhanced CYP3A4 mRNA expression in the FLC-5 hepatocellular carcinoma cell line [30] and in growth-arrested Hep G2/C3A spheroids [31]. Similarly, the induction of CYP3A4 activity by the PDMS plate (Figure 3) was consistent with data from perfusion culture using human primary hepatocytes [32]. Collectively, this evidence supports the notion that the PDMS-dependent oxygen supply is beneficial to spheroid culture, even when using a static culture method.

Hybrid spheroids containing equal numbers of cells and 20 µm-diameter hydrogel beads that form a network-like structure within the spheroid, exhibit elevated albumin secretion compared with conventional spheroids consisting only of cells [14]. Hydrogel beads are thought to reduce spheroid cell density and help to distribute oxygen internally, and the results reported here clearly demonstrate their effect in suppressing necrosis within the central region of spheroids compared with spheroids comprised of only cells (Figure 4). This effect extended from six to nine days when beads were used in combination with the gas-permeable plate. This effect suggests that the suppression of central necrosis by hydrogel beads occurs in an oxygen concentration-dependent manner. In contrast, there were no significant changes in cell growth, glucose consumption and lactate production due to the presence of hydrogel beads (Figure 2), nor on the mRNA expression and activity of CYP3A4 (Figure 3). Furthermore, the majority of the volumes of the spheroids were mid- (Hoechst+/pimonidazole+/Ki-67+) and high-oxygen (Hoechst+/pimonidazole-/Ki-67+) areas, which were unaffected by the embedding of hydrogel beads (Figure 5). It is therefore possible that these mid- and high-oxygen areas are the sites of cell growth, glucose/lactate metabolism and CYP3A4 expression/enzyme activity observed experimentally. An experimental system that facilitated the generation of larger low-oxygen areas, for example, a flow culture system where oxygen pressure could be finely adjusted, may be needed to demonstrate the effect of hydrogel beads more clearly.

In this study, we have addressed the possibility of improving oxygen supply to multicellular spheroids using PDMS and hydrogel beads. The effect of hydrogel beads is considered due to a plurality of factors, such as gas exchange other than oxygen, nutrient and waste exchange, and acquisition of cell-cell adhesion with polarity related-structures through gaps formed by hydrogel beads. Although this study has not addressed the effects of these factors, they should be considered in future studies. The hydrogel beads could also be coated with extracellular matrix components, since mesenchymal and vascular endothelial cells have different adhesion properties to cancer cells and could also be mixed into spheroids using our method. The basic system described here could therefore be further improved to more efficiently culture tumor tissues as well as multicellular spheroids.

## 5. Conclusions

The use of gas-permeable plates and embedded hydrogel beads effectively improves oxygen supply to multicellular spheroids. These observations are important to the development of systems for the maintenance of primary or PDX-expanded tumor tissues in vitro.

## References

[1] Tentler J.J., Tan A.C., Weekes C.D., Jimeno A., Leong S., Pitts T.M., Arcaroli J.J., Messersmith W.A., Eckhardt S.G. (2012). Patient-derived tumour xenografts as models for oncology drug development. Nat. Rev. Clin. Oncol..

[2] Siolas D., Hannon G.J. (2013). Patient-derived tumor xenografts: Transforming clinical samples into mouse models. Cancer Res..

[3] Hidalgo M., Amant F., Biankin A.V., Budinska E., Byrne A.T., Caldas C., Clarke R.B., de Jong S., Jonkers J., Maelandsmo G.M. (2014). Patient-derived xenograft models: An emerging platform for translational cancer research. Cancer Discov..

[4] Day C.P., Merlino G., Van Dyke T. (2015). Preclinical mouse cancer models: A maze of opportunities and challenges. Cell.

[5] Sacchi A., Mauro F., Zupi G. (1984). Changes of phenotypic characteristics of variants derived from Lewis lung carcinoma during long-term in vitro growth. Clin. Exp. Metastasis.

[6] Hausser H.J., Brenner R.E. (2005). Phenotypic instability of Saos-2 cells in long-term culture. Biochem. Biophys. Res. Commun..

[7] Kasai F., Hirayama N., Ozawa M., Iemura M., Kohara A. (2016). Changes of heterogeneous cell populations in the Ishikawa cell line during long-term culture: Proposal for an in vitro clonal evolution model of tumor cells. Genomics.

[8] Drost J., Clevers H. (2018). Organoids in cancer research. Nat. Rev. Cancer.

[9] Kondo J., Endo H., Okuyama H., Ishikawa O., Iishi H., Tsujii M., Ohue M., Inoue M. (2011). Retaining cell-cell contact enables preparation and culture of spheroids composed of pure primary cancer cells from colorectal cancer. Proc. Natl. Acad. Sci. USA.

[10] Neal J.T., Li X., Zhu J., Giangarra V., Grzeskowiak C.L., Ju J., Liu I.H., Chiou S.H., Salahudeen A.A., Smith A.R. (2018). Organoid Modeling of the Tumor Immune Microenvironment. Cell.

[11] Muraro M.G., Muenst S., Mele V., Quagliata L., Iezzi G., Tzankov A., Weber W.P., Spagnoli G.C., Soysal S.D. (2017). Ex-vivo assessment of drug response on breast cancer primary tissue with preserved microenvironments. Oncoimmunology.

[12] Nishikawa M., Kojima N., Komori K., Yamamoto T., Fujii T., Sakai Y. (2008). Enhanced maintenance and functions of rat hepatocytes induced by combination of on-site oxygenation and coculture with fibroblasts. J. Biotechnol..

[13] Nishikawa M., Yamamoto T., Kojima N., Kikuo K., Fujii T., Sakai Y. (2008). Stable immobilization of rat hepatocytes as hemispheroids onto collagen-conjugated poly-dimethylsiloxane (PDMS) surfaces: Importance of direct oxygenation through PDMS for both formation and function. Biotechnol. Bioeng..

[14] Kojima N., Takeuchi S., Sakai Y. (2014). Fabrication of microchannel networks in multicellular spheroids. Sens. Actuators B Chem..

[15] Motoyama W., Sayo K., Mihara H., Aoki S., Kojima N. (2016). Induction of hepatic tissues in multicellular spheroids composed of murine fetal hepatic cells and embedded hydrogel beads. Regen. Ther..

[16] Kojima N., Takeuchi S., Sakai Y. (2014). Engineering of pseudoislets: Effect on insulin secretion activity by cell number, cell population, and microchannel networks. Transplant. Proc..

[17] Kojima N., Takeuchi S., Sakai Y. (2012). Rapid aggregation of heterogeneous cells and multiple-sized microspheres in methylcellulose medium. Biomaterials.

[18] Tao F., Mihara H., Kojima N. (2019). Generation of Hepatic Tissue Structures Using Multicellular Spheroid Culture. Hepatic Stem Cells.

[19] Merkel T., Bondar V., Nagai K., Freeman B., Pinnau I. (2000). Gas sorption, diffusion, and permeation in poly (dimethylsiloxane). J. Polym. Sci. B.

[20] Nahmias Y., Kramvis Y., Barbe L., Casali M., Berthiaume F., Yarmush M.L. (2006). A novel formulation of oxygen-carrying matrix enhances liver-specific function of cultured hepatocytes. FASEB J..

[21] Arteel G.E., Thurman R.G., Yates J.M., Raleigh J.A. (1995). Evidence that hypoxia markers detect oxygen gradients in liver: Pimonidazole and retrograde perfusion of rat liver. Br. J. Cancer.

[22] Raleigh J.A., Calkins-Adams D.P., Rinker L.H., Ballenger C.A., Weissler M.C., Fowler W.C., Novotny D.B., Varia M.A. (1998). Hypoxia and vascular endothelial growth factor expression in human squamous cell carcinomas using pimonidazole as a hypoxia marker. Cancer Res..

[23] Sobhanifar S., Aquino-Parsons C., Stanbridge E.J., Olive P. (2005). Reduced expression of hypoxia-inducible factor-1alpha in perinecrotic regions of solid tumors. Cancer Res..

[24] Gerdes J., Schwab U., Lemke H., Stein H. (1983). Production of a mouse monoclonal antibody reactive with a human nuclear antigen associated with cell proliferation. Int. J. Cancer.

[25] Carmeliet P., Jain R.K. (2011). Molecular mechanisms and clinical applications of angiogenesis. Nature.

[26] Biel N.M., Siemann D.W. (2016). Targeting the Angiopoietin-2/Tie-2 axis in conjunction with VEGF signal interference. Cancer Lett..

[27] Nishida-Aoki N., Gujral T.S. (2019). Emerging approaches to study cell-cell interactions in tumor microenvironment. Oncotarget.

[28] Friedrich J., Ebner R., Kunz-Schughart L.A. (2007). Experimental anti-tumor therapy in 3-D: Spheroids–old hat or new challenge?. Int. J. Radiat. Biol..

[29] Hamon M., Hanada S., Fujii T., Sakai Y. (2012). Direct oxygen supply with polydimethylsiloxane (PDMS) membranes induces a spontaneous organization of thick heterogeneous liver tissues from rat fetal liver cells in vitro. Cell Transplant..

[30] Iwahori T., Matsuura T., Maehashi H., Sugo K., Saito M., Hosokawa M., Chiba K., Masaki T., Aizaki H., Ohkawa K. (2003). CYP3A4 inducible model for in vitro analysis of human drug metabolism using a bioartificial liver. Hepatology.

[31] Bavli D., Prill S., Ezra E., Levy G., Cohen M., Vinken M., Vanfleteren J., Jaeger M., Nahmias Y. (2016). Real-time monitoring of metabolic function in liver-on-chip microdevices tracks the dynamics of mitochondrial dysfunction. Proc. Natl. Acad. Sci. USA.

[32] Ortega-Ribera M., Fernández-Iglesias A., Illa X., Moya A., Molina V., Maeso-Díaz R., Fondevila C., Peralta C., Bosch J., Villa R. (2018). Resemblance of the human liver sinusoid in a fluidic device with biomedical and pharmaceutical applications. Biotechnol. Bioeng..

